# IoT and Engagement in the Ubiquitous Museum

**DOI:** 10.3390/s19061387

**Published:** 2019-03-21

**Authors:** Roberto Pierdicca, Manuel Marques-Pita, Marina Paolanti, Eva Savina Malinverni

**Affiliations:** 1 DICEA Universitá Politecnica delle Marche Ancona, 60131 Ancona, Italy; e.s.malinverni@staff.univpm.it; 2 CICANT, Universidade Lusófona (ULHT), 1700-097 Lisbon, Portugal; manuel.pita@ulusofona.pt; 3 Department of Information Engineering, DII, Universitá Politecnica delle Marche Ancona, 60131 Ancona, Italy; m.paolanti@staff.univpm.it

**Keywords:** IoT, space sensing, mobile sensors, museum visitor analysis, museum behaviour prediction, visitor attention, visitor engagement

## Abstract

In increasingly hyper-connected societies, where individuals rely on short and fast online communications to consume information, museums face a significant survival challenge. Collaborations between scientists and museums suggest that the use of the technological framework known as Internet of Things (IoT) will be a key player in tackling this challenge. IoT can be used to gather and analyse visitor generated data, leading to data-driven insights that can fuel novel, adaptive and engaging museum experiences. We used an IoT implementation—a sensor network installed in the physical space of a museum—to look at how single visitors chose to enter and spend time in the different rooms of a curated exhibition. We collected a sparse, non-overlapping dataset of individual visits. Using various statistical analyses, we found that visitor attention span was very short. People visited five out of twenty rooms on average, and spent a median of two minutes in each room. However, the patterns of choice and time spent in rooms were not random. Indeed, they could be described in terms of a set of linearly separable visit patterns we obtained using principal component analysis. These results are encouraging for future interdisciplinary research that seeks to leverage IoT to get numerical proxies for people attention inside the museum, and use this information to fuel the next generation of possible museum interactions. Such interactions will based on rich, non-intrusive and diverse IoT driven conversation, dynamically tailored to visitors.

## 1. Introduction

The digital revolution is changing the definition of the traditional museum, and opening the space for a wide range of novel visitor dynamics. The integration of technologies known as the Internet of Things (IoT) is expected to play a fundamental role in enabling new forms of content presentation and assimilation [1]. These in turn will allow for better human extended cognition [2], which impacts on user-experience by leading to improved knowledge acquisition and meaning construction, as well as leveraging social learning. The prevailing paradigm that defines human–museum interaction is based on the idea of a rather one-sided interaction, where all the meaning construction is solely on the side of the observer/visitor. Through the ongoing revolution, museums are fast becoming meaningful in new, more interactive ways—to the extent that they are turning into social learning spaces [3].

Museum directors and curators are becoming actively engaged in incorporating IoT into their spaces and curated exhibitions [4]. The reason for this is that the costs of such technologies are coming down. In addition, there is growing agreement that the correct adoption of IoT will become a differentiating factor in a highly competitive information ecosystem. There are several ways in which museums are tackling the current challenges posed by the digital revolution. An approach that is becoming very popular is to extend the range of museum “services” beyond preserving and displaying artworks to become places of leisure and education. One of the goals of such approach is to attract and retain new audiences, as well as to provide the means and resources to create more measurable engagement [5].

There is growing interest in the scientific community to understand how—through IoT—museums can engage in meaningful conversation with visitors, considering that, e.g., they are different from each other in fundamental ways. One of the key factors previously studied by other groups—and which was also the focus of the study presented here—has to do with the time people spend visiting a museum, or observing a specific element such as an artwork [6,7].

We followed this line of inquiry to study the link between IoT and length-of-visit, but framing this in connection with a third variable we consider to be critical: visitor’s *attention*. While attention remains difficult to define in concrete terms, we know a key feature of a working definition concerns the intentional decision of the mind to give salience to some object(s), at the expense of blurring others [8]. Attention is the essential resource needed for transferring any kind of information to the mind. After information transfer, attention can be considered “profitable” if it elicits internal information processing that has a measurable effect, such as learning something new that can be later recalled. A critical challenge for an “attention economy” in the context of this work is to measure—non intrusively—the amount of information transfer and processing that a museum elicits in a given visitor. Providing the means to measure attention non-intrusively would be an invaluable support for the museum of the future. Because of its inherent computational power, ability to measure different aspects of behaviour, and low demands on individuals, IoT will prove to be a fundamental key player for achieving this goal. For curators and directors, a reliable indicator of visitors’ attention would provide key support to decisions concerning, e.g., the cognitive complexity and size of exhibitions at the design stages; better planning for use of space vis-à-vis desired information flows, narratives, and so on [9].

We present a study that was carried out in the famous “Rocca di Gradara” museum located in Pesaro-Urbino, Italy. The foundation of this study was an IoT architecture we designed and installed inside the museum for collecting visitor data. This simple architecture consisted of a network of Bluetooth Low Energy (BLE) beacons placed inside the different rooms in the museum following a number of design and functional criteria. The Beacons were then paired with a proprietary mobile application that provides users with contextual information, while enabling us to gather visitor data. We processed the collected visitor data by performing statistical analyses aimed at getting baseline insights about visit patterns. These patterns were then used to derive a set of conclusions about visitor attention.

The goal of this study was to contribute to the methodologies that enable the use of both novel IoT architectures and suitable algorithms to derive indicators concerning visitor attention with a significant degree of confidence. While such an indicator of attention is likely to depend on many different factors and variables, in this work, we focused on a baseline study considering: (1) the patterns of choices individuals make regarding what to see in the museum—specific rooms in the case of this study; and (2) completing patterns of choices with the total time spent in each of the visited rooms. Furthermore, we looked at these variables in a dataset comprising the records we took of sparse and independent visits by single people. That is, we did not consider groups of any size, or concurrent visits. The idea behind this approach is to look at museum visits from an “atomic” level, and from this baseline build an increasingly more complex model in follow-up research. The resulting patterns of room-choices and time-spent can then be analysed in many ways, including factors such as space organisation, the architectural features of the museum space itself, how crowded the museum is at given times, days of the week or seasons, and so on.

The core contributions of the work presented here are: (1) an implementation of a real-world architecture based on the IoT model to collect and process user-generated data inside a museum in a reproducible manner; (2) a statistical account of how a network of sensors based on subject proximity may be used as a proxy for studying the patterns of individual attention in the context of a curated exhibition in a specific space; and (3) conclusions that provide strong motivating support for further exploration in this area towards enabling the ubiquitous museum to feedback in the design of its spaces, and contents, towards maximising the quality of visitor experience. This study thus constitutes a first step towards developing a new theory we can use to explain how to get good attention proxies and use them to create value inside the ubiquitous museum of the future.

The reminder of the paper is organised as follows: Section 2 provides a brief review of related works, adopting similar approaches, employing digital technologies, for understanding visitors’ museum experience. Section 3 describes the interdisciplinary methodologies used that constitute proposed approach and foundation of our studies, and provides details on the underlying technological architecture. In Section 4, we present an extensive evaluation of our approach, as well as a detailed analysis of data collected. Finally, in Section 5, we draw conclusions and discuss future directions for this field of research.

## 2. Background

A current key challenge for museums is to understand the patterns of interaction between its visitors and curated exhibits or the architectural features of its physical spaces. This is essential to determine the different mechanisms that can be used to attract attention and engagement effectively along the time-line of the visitor’s museum experience. Cognitive psychology has relied on empirical analysis to evaluate the factors and contexts that influence attention [10]. While there is empirically driven theory to measure attention in elementary perceptual tasks—both under control and experimental settings—there is no solid framework to study attention in complex scenarios that match museum experiences [11]. One of the differentiating domain specific factors introduced in recent literature is the concept of “Museum fatigue” [12]. Museum fatigue is a compound variable that determines phenomena such as cognitive overload. Such fatigue can be caused by spacial features such as light and air quality, the amount and nature of stimuli presented, failure to create engaging first interactions, the nature and flow of information presented to the visitor, and many other features. Some of these variables may affect many visitors in the same way, while others affect visitors with a diversity of cognitive styles and preferences deferentially. In any case, the outcome is always measurable as *reduced attention* and ensuing disengagement. If museums could detect low attention, without interfering with the visitor, e.g., by asking directed questions, then unobtrusive interventions aimed at improving the museum experience adaptively could be studied. Such interventions may be a key to start or re-ignite meaningful conversations between the museum and its visitors.

Different mathematically-derived indexes have been used to measure attention in museum visitors. One of them is the Diligent Visitor Index (DV) [9]. This index is calculated simply as the proportion of visitors that stopped at at least half of the museum elements (e.g., artworks). Another index (SRI) is calculated as the average total visit time divided by the size of the museum area that can be explored by visitors [9]. Other approaches rely on measuring various forms of engagement by groups of targeted visitors [13]. A recent trend uses tracking technologies that gather data, which can then be analysed to infer attention. See [14] for a comprehensive review. This survey and other recent works show that, while technologies that can be incrementally seen as IoT services have been developed during the last years, most of them propose new methods to support visitor interaction with the space. Little work has been done to understand and create theory on how to exploit user interaction data [15,16]. A key topic in this context is collaboration and co-design [17,18], with particular consideration to interaction [19] and the impact of different types of media [20,21]. A second line of research investigates how technology [22], and electronic guides can encourage learning [23,24]. Lastly, another topic of research focuses on the impact of visiting in groups, such as a families [25] with or without children [26]. Transversely to these areas, there is interest in understanding the use of (mobile) technologies and IoT to understand how visitors behave inside museum spaces [27]. Furthermore, we know that supporting visitors’ self-assessment inside the museum experience impacts how they interpret and recall it significantly [28]. A common limitation across all these approaches and perspectives is scalability and, subsequently, generalisation [29]. With regards to visitor behaviour, and user models, most relevant research has focused solely on matching the intentions and narratives designed by museum curators, and the interpretations of these by museum visitors [30,31].

Recent advances in sensor-based systems for studying social behaviour, such as the ones in social signal processing [32], participatory sensing [33], and opportunistic user context recognition [34], describe how automated sensing is a possibility to overcome these limitations. Few sensor-based systems have been adopted to study visitor behaviour inside fine-art exhibitions, particularly in connection to their actions such as choices of what to engage with [35]. Early works look at how monitor the movement of visitors in the rooms using Bluetooth data collected from mobile phones indoor localisation systems [36]. Such localisation data can be analysed, for instance, to encourage visitors to complete multimedia guides [37,38]. The limitation of these first works is scalability—these systems only cover the room that an individual is visiting in a given time. More recently, sensor technologies that include positioning and physiological data, along with entrance and exit surveys, have been used to study the cognitive reaction and social behaviour of individuals in an exhibition. The gathered data are analysed for classifying visitor experience into one of three categories: “the contemplative”, “the enthusiast”, and “the social experience” [39]. An analogous device devoted to measuring position and spatial orientation of individuals has been adopted to study the behaviour of people pairs in a museum. The work describes a system for pairs classification early in the visit into one of six classes, to provide socially-aware services to them, which are useful for growing their engagement with the exhibition [40]. Even in this case, the granularity of their data is not convenient to discern which zones visitors face. Lanir et al. proposed a system targeted towards the museum personnel with the aim to help them understand various behavioural patterns of visitors. They used a system based on radio signal (RFID) devices to detect their position [41]. Data coming from a multimedia museum guide have been used to predict visitor profile type into classes defined as “ant”, “fish”, “butterfly”, and “grasshopper” for personalising visitors’ guide overload [42]. Lastly, similar approaches have been adopted for virtual environments. In particular, localisation information is used to visualise users’ movements in a virtual museum. In [43,44], the metrics popularity, attraction, holding power and flows have been investigated for their visualisation. These works focus on visualisation of data without exploring deep data-mining techniques and data-filtering, and focus on simple exhibit-to-exhibit transitions without tackling longer sequences as paths. Crowd monitoring instead can be done by tracking the absolute position of each individual. However, this technique is a feasible solution in outdoor situations [45], where the GPS system can be exploited; for indoor scenarios, accurate localisation is still an open problem [46,47,48]. In fact, it is a well known problem that the localisation error increases significantly at the edges of rooms and in hallways—conditions that are often present in museum typologies [49].

The current state of the art shows a clear trend that will reshape the concept of museums. This trend involves the exploitation of IoT for providing services to the visitors, while using it also for getting user data that are translated into adaptive interventions. Indeed, we propose here the use the length of stay at a particular location as a baseline indicator for measuring the attention span with a view to its use in very significant improvements on visitor experience in museums.

## 3. Materials and Methods

In this section, we describe the scenario where the sensor network was implemented and used to collect visitor data. With regards to the sensor network architecture, it was conceived to meet the following criteria:

- enable a bidirectional communication between WSN and mobile devices;
- function for a long duration with minimal maintenance; and
- be compatible with customer grade mobile devices.

**Figure 1:**
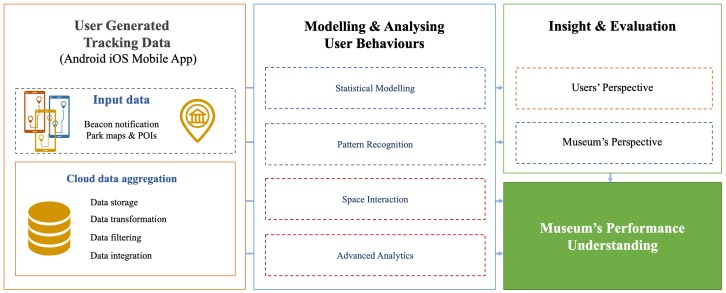
General overview of the research, from the collection of the data to their modelling, analysis and interpretation for planning purposes.

Section 3.1 defines the environment—in this case an indoor museum, where the sensor network was installed and data collected. Afterwards, in Section 3.2, we focus on the hardware components adopted for providing information and collecting data, specifically, *Estimote* beacons. This section ends with a description of Rocca di Gradara mobile application (Section 3.3) and the cloud based architecture of data collection. This work benefited from a deep analysis of the subject museum by experts. This allowed us to understand the physical space, and the nature of the curated exhibition used in our research, including the best beacon arrangement. Figure 1 shows a schema of our research approach.

### 3.1. The Real Environment: Rocca di Gradara Museum

Rocca di Gradara is the most visited museum in the Marche Region [50] located in the Pesaro-Urbino Province, in Italy, thus resulting a jewel of Italian fortified architecture. Over the centuries, it was reconstructed through several phases, until the last extensive restoration that took place between 1921 and 1923. The building has been marked with a strong imprint of medieval and neo-medieval style.

**Figure 2:**
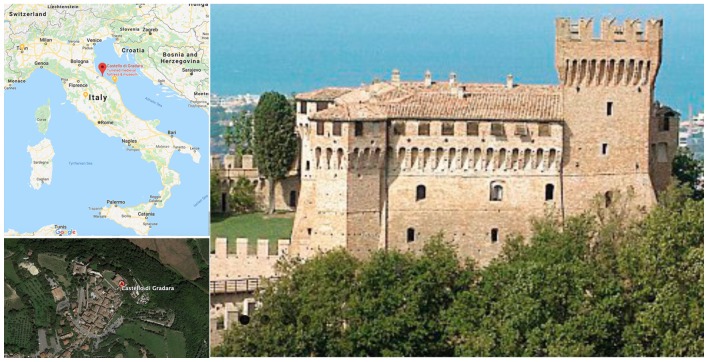
General overview of the museum. The view from the west side of the castle, image courtesy of Polo Museale delle Marche.

Figure 2 shows a general overview of the castle. In this picture, it is possible to perceive its quadrilateral layout with corner towers, as a typical example of fourteenth century military architecture.

The restoration program that includes the consolidation of the walls as well as the arrangement of the interior were carried out between 1921 and 1923 by the engineer Umberto Zanvettori. He recreated a typical style of residences from the Middle Ages and the Renaissance, which makes the environments that not only recall private residences but also the rooms of public palaces.

The theatrical and dramatic decor was carefully chosen by Zanvettori, who purchased numerous works of important value including antiques and precious fabrics and paintings. Its itinerary attracts thousands visitors per year. The museum can elicit multi-factorial interest because of its architecture, history, and the works on display. Thus museum curators are increasingly interested in understanding and improving the ways in which the museum starts and keeps conversation with visitors.

**Figure 3:**
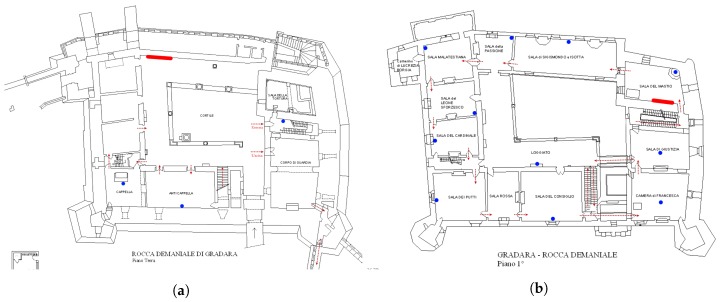
General arrangement of the museum: ground floor (**a**); and first floor (**b**) highlighting in blue the beacons’ locations and in red the position of two multimedia display.

The design of a standard visit is split into two levels. Visits start at the main entrance located in the courtyard. The visitor is conducted through fifteen rooms, following a set path. The implementation of the mobile application used in this work was conceived bearing in mind a straightforward guideline: the app must be part of a coupled system with the sensor network. The goal of this coupling was to track individual visitor entries to individual rooms with high precision, and without ambiguity. A map of the two floors highlighting the beacon positions is shown in Figure 3.

Through the different museum rooms, visitors retrace the Griffo’s and the Sforza’s families lives, as well as the Malatesta’s, the Borgia’s and the Della Rovere’s. It was in one of these rooms that on 1 September 1289 the Paolo and Francesca’s tragedy took place, an event that was immortalised by Dante in his Canto V of the Divine Comedy.

### 3.2. Network of Sensors

The sensors chosen for the installation are Estimote Beacons [51]. These are long-range location beacons based on BLE technology—a relatively new wireless technology developed by the Bluetooth Special Interest Group as a low-power solution, which may contribute to connect many devices, exploiting the IoT paradigm. The task of each beacon is to broadcast an encoded signal that identifies each of them to the mobile devices within a specified radius of influence. The device can measure the received signal strength intensity to infer, approximately, the distance to the beacon. Given their high frequency band of operation, their performances can be affected by the operational condition in which they are installed. To overcome this issue, beacon installation should meet the following requirements:

- Overlapping of signal between different sensors should be avoided.
- Beacons should be installed far from any source of noise.
- The placement of the sensors must allow the detection of all visitors who pass through the planned path.

Considering features of the museum, such as thickness of walls, furniture and others, we determined the best placement of the beacons in our sensor network by performing fine-tuning empirical analyses done in a laboratory and inside the museum.

### 3.3. Mobile Application

**Figure 4:**
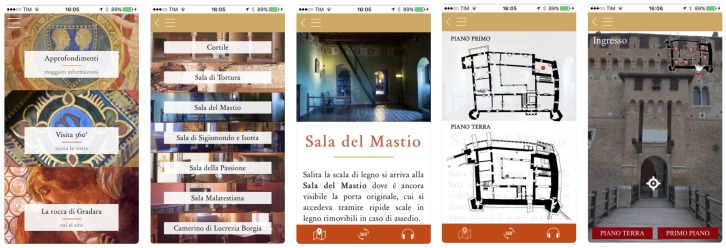
Screen shots of the app running. From left to right: The home page, the list of room, a detail of the room, the user geo-localised in one of the rooms and the panoramic virtual tour.

The Rocca di Gradara app was conceived to enhance the visiting path of the exhibition with the primary purpose of guiding the users among the rooms. Using room location detection, the app provides contextual information the current stage of a visit, leveraging the learning activity by the user. The main functions of the app are the real-time localisation and the contextual notification of the points of interest. Besides that, other functions increase the user’s experience, e.g., the audio-guide and the virtual tour, which enhances the way finding within the exhibition. A snapshot of the mobile application running is depicted in Figure 4.

### 3.4. Data Collection and Data Analysis

To gather user data, we deployed a cloud service, in which data are collected by following pre-defined rules. Once the mobile device is permanently within the operational range of the beacon, the application: (i) notifies the user with suggestions about possible routes; and (ii) sends position data to the cloud. The same criterion is adopted to register the activity of a device once the mobile device exits from this area of influence of the beacon. It is important to clarify some technological aspects related to communication issues. Since the building is made of heavy walls, the positioning of the sensors has been made to impede the decay of the signal. Moreover, since the app contains high quality multimedia files, the installation of a Wi-Fi connection was essential to guarantee their access. Finally, a caching system was developed within the app to send the data to the cloud even in the case of bad or no-signal.

Furthermore, the deployment was done performing empirical tests to verify that no overlaps occurred between beacons and that devices could be reached from all points of the visit. In this way, it was possible to minimise errors for the purpose of collecting more reliable data. To summarise, we collected data coming from beacons sensing device-id entries and exits for each of the twenty rooms containing curated content in the museum. This resulted in a dataset containing records for fifty-six different individual, non-overlapping visitors. The data record for each visitor consists simply of a sequence of tuples. Each such tuple contains a *room name*, *device-id*, *entry timestamp*, *exit timestamp*, and a computed *visit duration*.

All information of the user is stored using a unique identifier, which is the Media Access Control (MAC) address of each device (used as UUID). At the time of the experiment (2017), gathered data were considered untied from any personal identification by means of an anonymisation process. This fixed MAC address in fact cannot be linked to any personal information such as names or phone numbers, allowing for the collection of different types of information only from the mobile device. Furthermore, no personal data are stored by the systems deployed. In this way, we have not been able to associate the data with any specific participant. However, with the advent of the new GDPR, MAC address is considered as “linkable information”. Thus, a new version of the app will be deployed considering this legal obligation.

### 3.5. Statistical Methods

We obtained a matrix dataset *S* that has twenty rooms and fifty-one subjects. The raw dataset had 56 subjects, but five were removed due being either records of the experimenters, or records of subjects whose total visit duration was less than one minute. In the dataset matrix *S*, each element $S_{i,j}$ thus represents the visit length to room *i* by subject *j*. This $S_{i,j}$ is the sum over all visit intervals determined by possible multiple re-entries by subject *j*. We computed *total visit length* (over the different rooms) for every subject *j*. For each room *i*, we computed the total number of unique visitors it had. When the number of visitors of a room was less than 10% of the total number of subjects, the room data were eliminated from the dataset, and all subsequent analyses. This led to the elimination of six rooms, as explained in Section 4. For the remaining 14 rooms, we looked at the distribution of visit lengths and determined that 13 out of the 14 distributions are not normally distributed (Jarque–Bera test α=0.01) [52]. We then computed the median time subjects spent in each of them. While doing this, we removed far outliers based on the 1.5 Inter-Quartile rule. Then, we computed the number of individual room visits in 1-min cumulative intervals starting from 1 to 8 min. Qualitative analysis of content complexity for each room (see Supplementary Materials SI-2) determined that, for a visitor to assimilate minimal content, a visit lasting a minimum of three minutes is required. Therefore, any room visit lasting less than three minutes was labelled as an *impression*, and as a *consumption* otherwise. We computed the proportions of room visits that can be considered impressions and consumption for the three-minute qualitative threshold. To determine differences between rooms in terms of length of visit distributions, we computed all pairwise Mann–Whitney–Wilcoxon two-tailed statistics [52] to determine median differences. Since this amounts to performing multiple tests on the same data, we corrected the obtained *p-values* using false discovery rate (fdr) = 25% [53]. We performed Principal Component Analysis (PCA) [54] on the matrix *S* relating rooms (variables) and visitors (individuals) via visit lengths in minutes. The resulting principal components were represented as a matrix of factor loadings. For each principal component, we selected loadings for variables with magnitude p≥0.3. While there are no hard rules on what the cut-off point is for acceptance of loadings describing a PCA factor, the general agreement for sparse matrices and relatively small sample sizes is around p=0.3 (see [55]). We then confirmed the statistical correlational association between loadings and their specific principal component (Shapiro–Wilks Test [52] with α=0.05). Since our sample N=51 was relatively small, we performed further tests to find out if the PCA results were statistically significant and robust. The method used was a non-parametric bootstrap (see [56] and references therein) analysis in which we: (1) re-sampled N=51 individuals from the original data randomly with replacement; (2) performed PCA on the re-sampled matrix and stored the loadings originally selected for each component; (3) repeated this process for R=1000 samples; and (4) computed the 95% confidence interval (CI) for the median value of each loading using the appropriate technique for this scenario described in [57]. We accepted the loading as being statistically significant with α=0.05 either if the original loading falls within the corresponding bootstrap CI or if the CI low bound magnitude was p≥0.3.

## 4. Results

The goal of this work was to explore baseline behaviours in a group of people who interacted with a curated museum exhibition. As explained previously, this exhibition was enhanced with a Bluetooth beacon sensing network. Our analysis was based on: (a) the rooms people chose to visit; and (b) the time they spent in the different rooms. The key finding was that the vast majority of people spent very little time in the museum as whole, but following distinct visit patterns with moderate to high room selectivity. In Section 5, we elaborate on what this may mean in terms of subject attention span issues vis-à-vis the nature of the specific curated exhibition used for the experiment, and the future implications of IoT in its general context. The results are presented as follows. We started our analysis looking the the entire subject (visitor) timelines. First, we reported on the distribution of the total time subjects spent in the museum. Then, we contrasted this information with an analysis on the number of rooms subjects chose to visit. For the second part of our analysis, we shifted to the analysis of what happens in each of the rooms. We report on numbers of unique visitors, as well as the distributions of length-of-visit in each room. In particular, we present statistical pairwise comparative tests for the differences between length-of-visit distributions across different rooms. In the third stage of our analysis, we looked at how to separate individual room visits into classes determined by a qualitative threshold in visit length. One of these classes captured visits that were so short that they could only leave an *impression* in the visitor, not being successful in passing the curated information contained in the rooms and artworks to them. Conversely, visits that fell into the other class—*consumption*—allowed for sufficient time to acquire information that could be recalled in a later post-test. In the last stage of our analysis, we uses PCA on the matrix that relates visitors to rooms via length-of-visit to determine whether there were defined visit patterns captured as linearly independent meta-variables (features).

### 4.1. Data Pre-Processing

We collected data on the activity of fifty-six subjects who visited the museum’s curated exhibition freely while carrying an active Bluetooth device. We found that the original dataset contained records corresponding to the experimenters, as well data from subjects with close to no activity (total visit duration less than one minute). These entries were removed and we were left with a reduced dataset containing the activity of fifty-one unique subjects. Recall that subjects’ mobile devices were tracked using proximity by the beacons installed in the museum to detect entry to and exit from each individual room. The data returned by the beacons could be parsed into date and time data structures. The timestamp has minute-level precision, thus reason entries and exits happening in the same minute were recorded as lasting half a minute. On the other hand, we truncated the duration of individual room visits when records showed them lasting more than one hour, thus keeping the maximum room-visit duration one hour. The truncated individual room visits amounted to 1.3% of the 775 individual room visits we recorded. This truncation was implemented as a correction for possible beacon failures when recording exit times in a small number of cases, and to improve inferences made from the figures presented here. Given its small prevalence in the dataset, we do not expect any significant impact on the statistical analysis presented here caused by truncation.

### 4.2. Total Visit Length and Room Coverage

**Figure 5:**
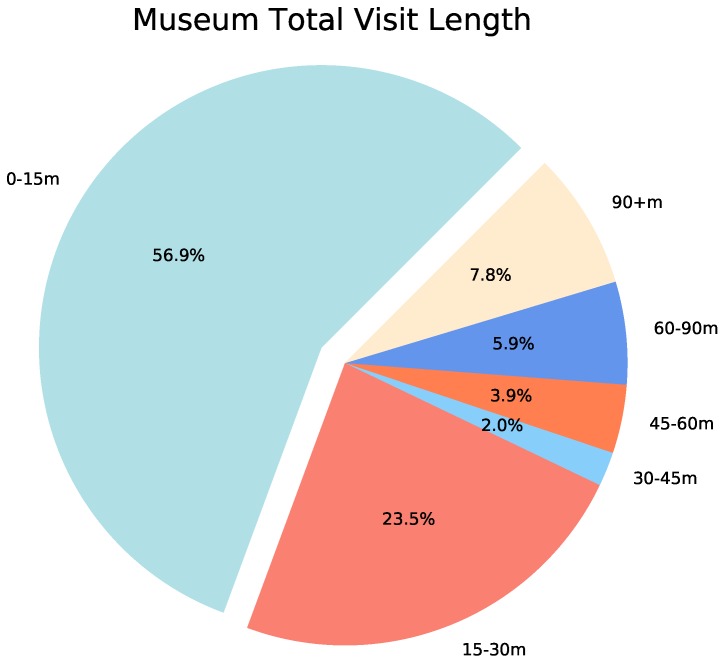
Distribution of individual total visit length. External labels correspond to time intervals in minutes, while inner labels correspond to proportion of visits lasting a a time period within the matching interval. The vast majority of subjects (80.4%) completed the entire visit to the museum in less than half an hour. Significantly fewer visits (13.7%) had a total duration of 60 min or longer.

The first exploratory statistical analysis of the collected data revealed that most subjects followed a visit pattern characterised by: short-length visits to a relatively small number of rooms. Figure 5 shows the distribution of entire visit length binned in six different fifteen-minute intervals. The vast majority of visits had extremely short duration considering the exhibition contains twenty different rooms, each of which is cognitively non-trivial. Indeed, 80.4% of the visits to the museum lasted less than thirty minutes, and a very small number of visits lasted more than one hour (13.7%), out of which 7.8% lasted over 90 min.

**Figure 6:**
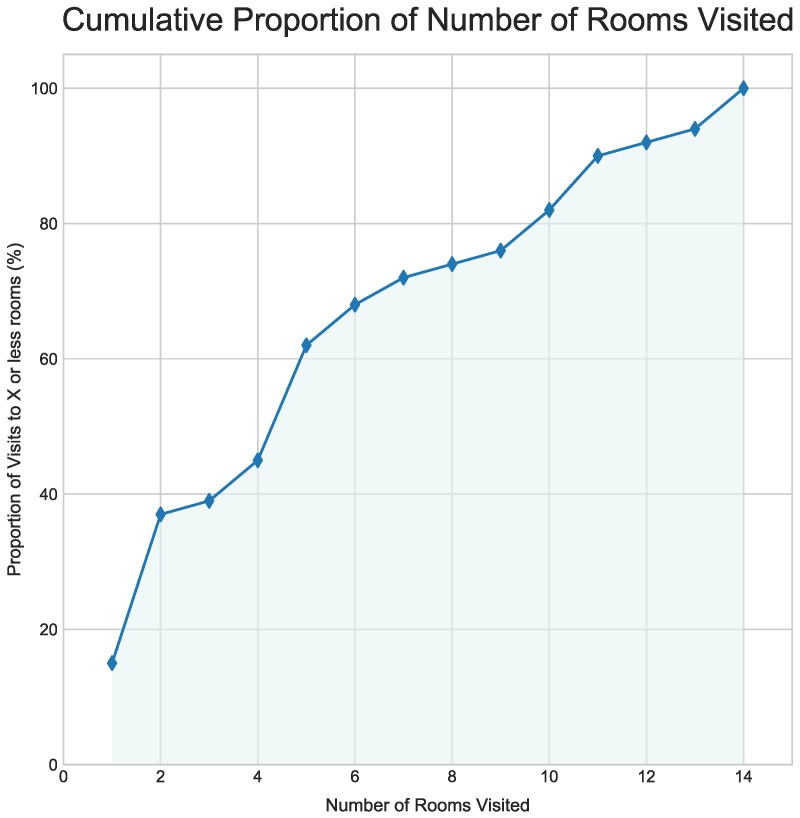
Cumulative proportion of number of rooms visited. The number of visited rooms varied from one to fourteen (out of twenty possible options). The average person visited between four and six rooms. In favour of high selectivity, notice that 40% of the subjects visited up to three rooms, and that the majority of this segment actually visited only two rooms. However, 60% of the subjects were moderate in selecting between four and fourteen rooms.

Concerning room coverage, we found that subjects used their time to visit 4–6 rooms on average—out of the twenty different options available to them (see Figure 6). Notice in the figure that around 75% of subjects visited up to eight rooms. This means that the average audience was never exposed to more than half of the content available for consumption in the exhibition. The remaining 25% of the subject population chose to visit between nine and fourteen rooms. No visits to fifteen or more rooms were recorded. These observations suggest that subjects were moderately to highly selective about the rooms they visited. In what follows, we elaborate on subject–room selectivity and the possibility that it is implicated in specific visit patterns. The next step was to look at the existing variability in the number rooms visited and the specific rooms chosen.

### 4.3. Visits to Individual Rooms

**Figure 7:**
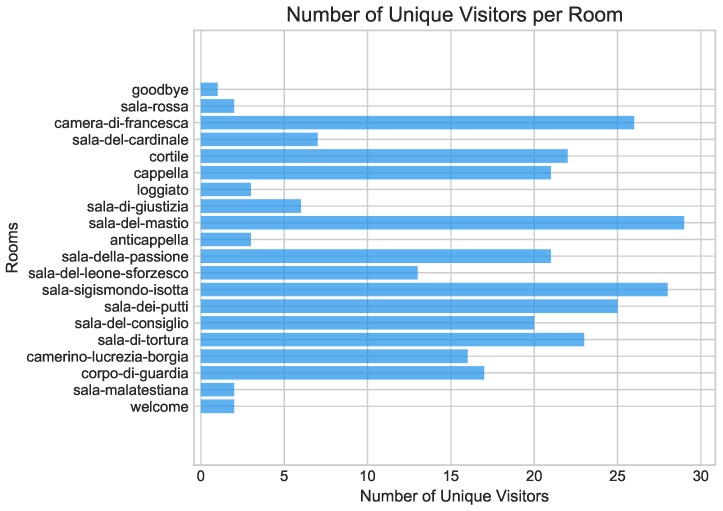
Number of unique visitors per room. In this figure we look at each room separately, showing the number of unique visitors each of them had. Four of the twenty rooms had 50% or more of the visitors, while six rooms had less than 8%. These latter six rooms were removed from subsequent analyses. Concerning the ongoing story about selectivity, notice that there are four rooms that are favoured by visitors, and that there are ten rooms that received a sizeable proportion of visitors.

The observation that most total visit lengths were very short, along with apparent moderate to high room selectivity, led us to explore visitor activity for each room next. Figure 7 depicts the number of unique visitors recorded for each of the twenty museum locations in which beacons were installed. The specific goal of this second analysis was to explore the choices made by different subjects with regards to what rooms to explore. This exploration allowed us to study room selectivity in more detail. Four rooms appeared as the most attractive with over 50% of the subjects having visited them: *Sala del Mastio, Sala Sigismondo Isotta, Camera di Francesca* and *Sala dei Putti*. Notice also that six of the exhibition rooms had fewer than five visitors: *Welcome, Sala Malatestiana, Anticapella, Loggiato, Sala Rossa* and *Goodbye.* Since the activity for each these six rooms corresponded to less than 8% of the total number of visitor activity, we removed them from subsequent analyses. The reason for this removal was that, with such small representation in the space of visits, these rooms were highly unlikely to be part of any significant visit patterns we may uncover in the subsequent analyses. After this removal, the remaining rooms with the fewest visitors were: *Sala di Giustizia* and *Sala del Cardinale*.

**Figure 8:**
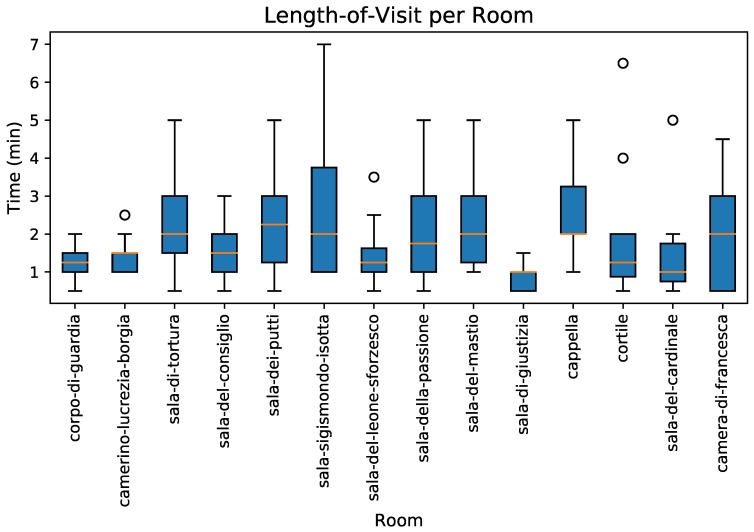
Length-of-visit distributions per room (T). Rooms could be roughly classified into two groups according to the time subjects spent in them. One group comprised rooms for which visitors spent one minute on average, with little variation, while the other group contained rooms for which the T median was between 1.5 and 2 min. They corresponded to distributions that also had larger dispersion (longer bars).

To get a more thorough picture of the interest each room elicited in the subjects—an important factor determining room selectivity—we looked at the length-of-visit variable, henceforth denoted by T. Figure 8 depicts the T distributions for individual rooms. Notice that this figure shows the fourteen rooms that were kept for further analysis. The lowest T medians were found for *Sala di Giustizia* and *Sala del Cardinale*, which were also the rooms with the fewest visitors that were kept for analysis. The median length of visits for the remaining rooms was also low, varying between 1.5 and just over 2 min. All four rooms with most visitors mentioned above had the highest median visit length—that is, 2 min or longer. *Sala di Tortura* and the *Capella* emerged in this analysis as rooms that, while not having the highest numbers of visitors (yet both had more than twenty visitors), attracted relatively long visits, with a median T of 2 min or longer in both cases.

To find how significant T differences were over all possible pairs of rooms, we performed pairwise comparisons using Mann–Whitney–Wilcoxon U test. This non-parametric statistical test was chosen after verifying most T distributions in the dataset do not pass a normality test, with the exception of $T_{\mathrm{cortile}}$. Given the sample sizes (N<25), the normality test used was the Jarque–Bera test (see Supplementary Materials SI-3). Since there were 14 distributions T, we performed 142=91 two-tailed tests. The obtained *p-values* were corrected using *False Discovery Rate* (fdr)=25% with critical *q-value* set to q=0.05. The resulting table is available in Supplementary Materials SI-4).

Unsurprisingly, the outcome of the statistical tests was that people spent significantly different lengths of time in *Sala de Giustizia* and *Sala del Cardinale* compared to in other rooms. These were the lowest “performing” rooms in terms of length-of-visit left in the analysis after removing the six rooms with worst performance. Conversely, the time people spent in *Sala del Mastio* and *Sala Sigismondo Isotta* were statistically different from time spent in most other rooms. These corresponded to the two rooms that attracted the most, and longer visits.

### 4.4. Impressions vs. Content Consumption

One of the important goals of a curated museum exhibition is to offer visitors the possibility to, e.g., build meaningful experiences or acquire useful information. Lacking specific interaction information, the time people spend in a well-defined space can be used to infer *how much* construction of an experience or information acquisition took place. This is particularly true when the time spent in these places is very short, indicating—with a large degree of confidence—that no complex experience that could be later recalled took place. Longer stays can only be used as evidence that information was *probably* consumed. Definitive confirmation of consumption would require post-visit recall tests.

**Figure 9:**
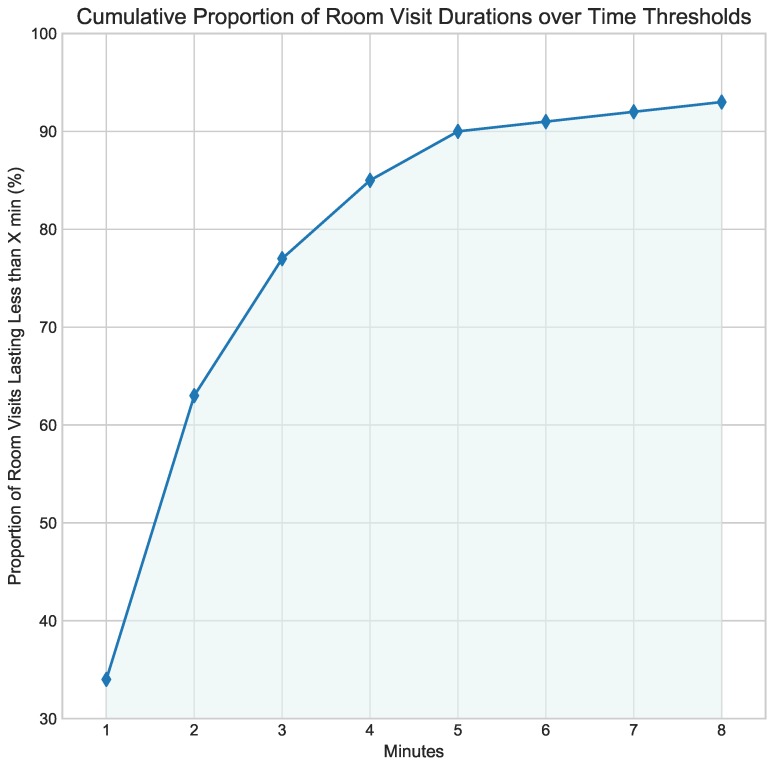
Cumulative proportion of room visits over time thresholds. A qualitative analysis of the curated content inside each room determined that a visitor would need at least three minutes for minimal content consumption. Our data show that approximately 75% of room visits could only be classed as *impressions.*

We performed a qualitative cognitive analysis of the curated spaces and content that make up the museum exhibition. This analysis yielded a threshold of three minutes for the average time a visitor of a room would need to assimilate *minimal* information about its contents. See Supplementary Materials SI-2 for details of this qualitative analysis. Subjects who spent less than three minutes in a room *X* were thus classified as people who got an *impression* of *X*. This means that a person who has an impression of a given room is unlikely to pass a recall test in which they are questioned about the curated content in that room. Conversely, longer visits were increasingly considered as likely information *consumption* events. Figure 9 shows the cumulative proportions of individual room visits over discrete time in minutes. Out of all the individual room visits on record, over 60% lasted two minutes or less. Considering the three-minute qualitative threshold separating impressions from consumption, close to 80% of room visits were indeed impressions.

**Figure 10:**
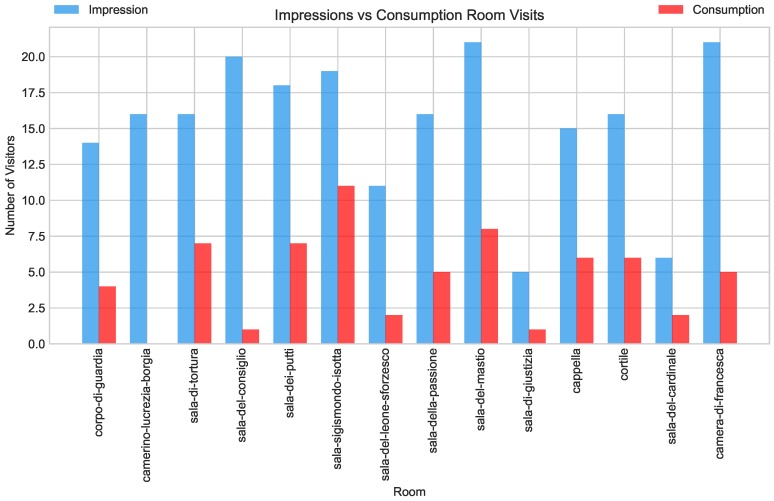
Room visits considered impressions or consumptions using a three-minute threshold. Blue bars represent number of visits that lasted less than three minutes, while red bars represent the opposite. From previous analyses, we know that most visits stayed below four minutes, which means that the red bars represent both shallow consumptions and the 15% of visits lasting beyond four minutes.

Another view of the impressions vs. consumption analysis is depicted in Figure 10. Notice that, while impressions dominate the space of visits, the four rooms with most visits had a larger proportion of consumption visits—even though the majority of these remained close to the three-minute threshold. *Sala Sigismondo Isotta* and *Sala del Mastio* stand out as the rooms in the exhibition that attracted most visitors, for longer periods.

### 4.5. Principal Component Analysis

In the final stage of the statistical analysis, we performed Principal Component Analysis (PCA) on the matrix *S* that relates the fourteen rooms and the fifty-one visitors via length-of-visit in minutes. The goal of performing PCA was to determine if there were linearly separable visit patterns—or visit modalities—that could be understood as interpretable meta-variables after dimensionality reduction. Such meta-variables could, in principle, inform adaptive action strategies for mobile device interventions aimed at improving the quality of visits.

#### 4.5.1. Visit Patterns

**Figure 11:**
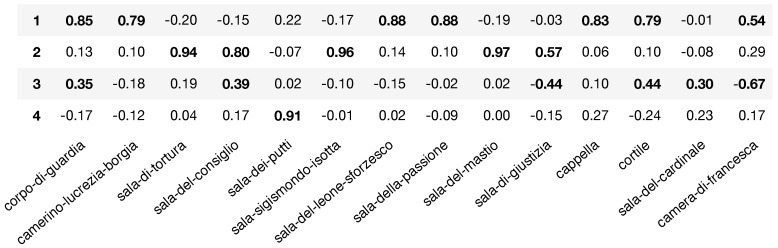
The PCA analysis of per-room length-of-visit data captured four meta-variables that described 75% of the variance for the entire visitor dataset.

Our first finding is that the first four components obtained from the PCA described 77% of the variation in the entire dataset. The PCA factor loadings are depicted in Figure 11. For details on the statistical methodology, see Section 3.5. We set a magnitude threshold p=0.3 to decide what room loadings to consider when interpreting a meta-variable obtained from the PCA. We then performed Shapiro–Wilk tests to obtain p-values that determine the significance of the correlational association between the chosen loadings and each PCA component. All the chosen loadings for every component were found to be strongly associated with their corresponding PCA components (see first three columns of Supplementary Materials SI-6). The first principal component explained 33% of the observed variation in choice of rooms and time spent in each for the entire dataset. The underlying pattern correlated room-choice and time spent in the following seven rooms: *Corpo di Guardia, Camerino Lucrezia Borgia, Sala del Leone Sforzesco, Sala de la Passione, Capella, Cortile* and *Camera di Francesca*, with no negatively loaded rooms. Considering this first component, and its positive loadings, the museum could set a baseline expectation that a regular visitor will spend time in a subset of the aforementioned seven rooms. The second principal component described about 27% of length-of-visit variability in the original data. This component was positively loaded by: *Sala di Tortura, Sala del Consiglio, Sala Sigismondo Isotta*, *Sala del Mastio* and *Sala di Giustizia.* No strong negative loadings were found for this second component. Here, we found a component that captured the visit pattern that favoured the two most visited rooms in the exhibition: *Sala Sigismondo Isotta* and *Sala del Mastio*. This visit pattern also corresponded to people who tended to spend more time in the rooms they visited. The third PC described around 9% of the dataset variability. This principal component had positive loadings for *Sala di Guardia, Sala del Consiglio, Cortile* and *Sala del Cardinale*, and significant negative loadings for *Sala di Giustizia* and *Camera di Francesca*. This component thus represented a small segment of visitors who followed a visit pattern characterised by lack of visits to *Camera di Francesca* and *Sala di Giustizia*, while favouring visits to rooms such as *Cortile*. The fourth principal component—which described about 8% of the dataset variability—associated strongly with visits to *Sala dei Putti,* with no strong positive or negative correlations to other rooms.

We performed a bootstrap analysis based on the non-parametric techniques discussed in [56] (and references therein) to determine the statistical significance and robustness of the visit patterns we identified through PCA. This involved the generation of R=1000 random samples derived from the original data (with replacement) and performing PCA for them all to obtain distributions for the relevant loadings identified in the original PCA. See Section 3.5 for method details. The result of this analysis is in Supplementary Materials SI-6 (last two columns). The 95% CI for the median value of relevant loadings: (1) confirmed that the first two principal components, which were the most descriptive visit patterns we found in terms of explanatory power, were robust and statistically significant; and (2) all of the loadings for PC3, which had low explanatory power (accounting for 9% of observed variation), were found to be unstable and not statistically significant; and (3) while the single relevant loading for PC4 was not contained in the CI obtained via the bootstrap, the estimated population median value remains above the 0.3 threshold ($CI_{\mathrm{pc}4}=\left(0.46,052\right)$ see Supplementary Materials SI-6). The observed instability in the PC3 bootstrap distribution might result from various factors. The most likely explanation is that PC3 was not linearly separable in general, unlike PC1 and PC2.

#### 4.5.2. Individual Visits

After analysing PCA loadings to characterise the features of visit patterns, we turned to the individual scores obtained from the PCA. Supplementary Materials SI-5 contains a table and visual representation of the scores for each of the fifty-one visitors across the four dimensions we kept after performing PCA. Useful conclusions from dimensionality reduction techniques such as PCA could be derived by looking at the scores for individuals. The idea was to determine the extent to which individuals fared much higher in one PC than others (thus being highly skewed), or whether, instead, they were best represented as linear combinations of the PCs where significant coefficients for different PCs are present. If the population turned out to be very skewed, it would be possible to classify individuals according to the specific PCs they were skewed to based on their specific scores. In this case, it would also be possible to compute the proportion of individuals that fell into each class (PC). However, in the case of our dataset—as expected—we found that most visits were described as moderately skewed linear combinations of the four PCs. There was a clear tendency for higher coefficient contribution from PC1—indeed 70% of individuals had their highest coefficient in this PC, but most of these also had a sizeable contribution of from the other PCs, especially PC2. Supplementary Materials SI-5 contains a visual representation of the individual scores that allowed inspecting the way in which visits were linear combinations of the four PCs easily. There were only a few cases of individuals who were strongly skewed to a single PC, out of which just one individual could be considered as belonging to one single PC class. These individuals are Visitors 32, 33 and 34 in the table and corresponding figure in Supplementary Materials SI-5. The visit pattern of Visitor 32, for instance, was mostly correlated with PC2 (score 10.69) but also PC1 (score 3.27); Visitor 33 could be classed as a prototypical example of PC4; and Visitor 34 was mostly PC1 with some PC3.

## 5. Conclusions and Future Works

In this work, we support the use of spacial sensor networks within an IoT framework to gather information about how people experience museums. This study was both preliminary and minimal in nature. One of the main contributions is to provide evidence of the relevance of IoT based technology to obtain user data, analyse it and leverage the scarce resource of human attention through carefully crafted interventions. Here, we focused on the first component, namely setting up a sensor network, gathering visitor data (choice of rooms, and time spent in each) and deriving features that describe how people visit this specific museum setting. This study was constrained to single visitors with no overlapping visit paths.

A big problem in this context was that attention is very hard to measure, especially in a non-intrusive manner. The area of opportunity was thus to be able to produce reliable estimators that can be successfully used for guiding interactions between museums and visitors in a rich, diverse and adaptive manner. We claim that IoT is a crucial part of any successful framework that solves this problem.

The goal for the visitor is to have relevant, appropriate and timely feedback that brings their attention into focus. For example, this feedback can be used to: (1) plan the entire visit in advance, or decide the next step in a visit based on information provided by the museum; and (2) manage individual expectations, get tested or exposed to background information, and explore the museum keeping a comfortable cognitive load. In this way, it can: (3) help visitors make useful connections between information elements in the museum’s narrative and prior visitor knowledge; and (4) keep a record of the visit for future review, production of social network content, future recommendations and indeed many other benefits. For the museum, the aim is to use solid indicators that measure: (1) the extent to which different visit patterns emerge, and how they can impact on the narrative goals of a given exhibition or space management; (2) what and how much information gets transferred to visitors (to infer selective attention); and (3) evidence pointing to differences in the ways content is consumed and interpreted by different people/groups and how this may feedback on the strategies the museum uses to engage visitors. A long term goal of adaptive museum conversations is to use the obtained information in a way that allows the museum to reconfigure itself dynamically. This would allow the museum to change its configuration—almost in real time—to adapt to the nature of specific groups visiting it. Such knowledge can also be used to recommend connections among people who share similar interests and preferences for how to consume content. Another benefit will be the evolution of conversation between individuals and the museum space. This conversation will have a sense of goals, background, and context, and will adapt to what a specific visitor is doing instead of using a “one size fits all” approach. Clearly, the degree to which these goals can be achieved—both for the museum and its visitors—will depend on various layers of individual consent to user data privacy and assurances concerning data protection.

### 5.1. Short, But Not Random, Attention Span

They key finding of this work is that, even for a small sample studied in a relatively limited setting, we found evidence **of very short attention** in the subject population visiting the museum. However, it was surprising to find that—even though length of visit was extremely short—**visit patterns were not random**. The sequences of chosen rooms and the time people spent in each of them followed clearly distinct patterns or modalities. Indeed, we uncovered four distinct visit patterns in this specific dataset, three of which were found to be statistically significant based on resampling techniques. The first describes visitors who select between three and six rooms—out of twenty—with a strong tendency to choose from a subset of seven rooms (see first component of PCA analysis in Section 4.5). The second visit pattern describes an interesting type of visitor who is moderately selective, and who has a strong tendency to visit the rooms that were visited by most people, for longer, namely sala Sigismundo Isotta and Sala del Mastio. For the remaining components, we found groups of people who appear to be highly selective in the sense of visiting a very small subset of the available rooms. This group had a strong tendency to favour visits to sala dei Putti (in one of the PCA components), while avoiding camera di Francesca (which for other visit patterns is an active room).

With this knowledge, it becomes possible to analyse visit patterns as well as specific information about, e.g., how long people spent in the different rooms. Such analysis can be framed in the context of the qualitative three-minute threshold that was used to divide impressions from consumptions. The results obtained from such analysis would make it possible to create a dialogue between the museum and a future visitor. The goal is to ask a small number of questions to quickly match a new visitor to one of the known visit patterns. Information about the visit pattern could then be used to guide the recommendations provided by the museum’s mobile app to the specific user. Clearly, such dynamic would lead to a constant revision of the internal model the museum has of its users, and the museum would become better at guessing the best visit pattern for a given individual as it gathers more data from different people. In addition, the same informational dynamics can be used by the museum to evaluate the reasons some spaces and narratives fail to engage visitors.

### 5.2. Limitations and Future Work

One limitation of this study was the total reliance on a Bluetooth-based beacon sensor network. Additionally, we were unsuccessful in convincing a larger number of visitors that downloading the mobile app used for the study would be worth it for them. Because of these two limitations, the number of visitors carrying a mobile phone that was on during the entire visit, had Bluetooth active, and had installed the mobile app was much smaller than the total number of visitors the museum had in the data collection period. Indeed, out of the people who downloaded the app, the proportion of visitors that we tracked was 25%. This led to a sparse dataset. That is, this study did not allow us to study patterns where crowding in rooms or around pieces could be studied. While this can be seen as a limitation—and it certainly is for many goals—in the case of this specific work, this small sample provided us with a dataset that allowed for the study of baseline behaviour in a minimal setting. From this basis, we can begin to study how phenomena such as crowding or other factors disrupts the visit patterns we encountered.

In future studies, we will expand the sensor network to include Wi-Fi as well as Bluetooth. We will also design a clear strategy to motivate visitors to download and engage with the museum’s app. That way, we expect much larger datasets for this museum. This will allow the confirmation or revision of the visit patterns reported in this paper. Importantly, a followup study will include an experimental design that allows us to study a range of factors affecting attention measurable via IoT. This way, we expect to gain a deeper understanding of how IoT enabled conversations between the museum and its visitors can result in better information consumption inside the museum. In future work, we will also explore working with targeted audiences, such as experts or occasional visitors and measure post-visit variables such as level of satisfaction and recall, leveraging the mobile application.

### 5.3. Final Remarks

Recent emerging technologies—together with their ubiquitous presence in our daily lives—represent a turning point in the way in which human behaviour analysis is performed. Besides the ubiquitous nature of sensors, the growing computational capability of mobile devices makes it possible to collect large datasets of human behaviour at high frequencies, sometimes even in real time. However, despite the widespread use of such data collection technologies, the analysis of visitor behaviour in art museums has not advanced much. Many art curators and museum managers still prefer a pen-and-pencil strategy based on visual observation or direct interviews with visitors. This subjective, intrusive approach, while to an extent was useful in the past, will become more of an obstacle and a setback for museums in the era of hyper-connected societies. The clear need is for a data-driven approach that provides principled measures and information to support design policies and improvement of the services provided within by museums. The methodological approach presented in this paper is for practitioners and curators to deepen their understanding of their visitors and to improve the overall quantity and quality of services offered. The core idea of our preliminary framework was to get a baseline insight about how IoT could be minimally used to make inferences about attention inside the museum. Using our results as a foundation, the next step is to improve on the mobile app and the entire sensing architecture so that it becomes useful in a bidirectional way.

## Supplementary Materials

The following are available online at https://www.mdpi.com/1424-8220/19/6/1387/s1. SI-1: DataSet; SI-2: Content Complexity; SI-3 Normality Tests for Individual Room Distributions (Jarque Bera); SI-4 Central-Tendency Pairwise Room Differences Mann-Whitney-Wilcoxon FDR Corrected for multiple tests; SI-5: PCA scores for the individuals; SI-6: Statistical significance of PCA factor (PC) loadings.
