# Supplementary material for: IoT and Engagement in the Ubiquitous Museum
Source: Sensors (Basel). 2019 Mar 21;19(6):1387. doi: 10.3390/s19061387 (PMC6470879; doi:10.3390/s19061387)
Supplement: Supplementary file 1 [file sensors-19-01387-s001.zip › Supplementary_Material/SI-1_Dataset/SM1_Notes.rtf]

Supplementary_Material_1 - SM1Beacons_Dataset is a csv file containing the dataset used for the experiment. Each row is the MAC address of the single device, columns represent enter and exit time into and from the beacon radius of influence, the time stamp and the location of each beacon.
