# Supplementary material for: IoT and Engagement in the Ubiquitous Museum
Source: Sensors (Basel). 2019 Mar 21;19(6):1387. doi: 10.3390/s19061387 (PMC6470879; doi:10.3390/s19061387)
Supplement: Supplementary file 1 [file sensors-19-01387-s001.zip › Supplementary_Material/SI-2_Content_Complexity/SM2_Notes.rtf]

Supplementary_Material_2 - SM2The file Areas_Gradara and Areas_Gradara_1 are csv file used for the qualitative analysis of content complexity for each room. It has been used to determine that for a visitor, to assimilate minimal content, a visit lasting a minimum of three minutes is required. The rows represent the rooms for the ground and first floor, the columns contain an ID number, the squared meters, the connection to other rooms and their content complexity.
