# Supplementary material for: IoT and Engagement in the Ubiquitous Museum
Source: Sensors (Basel). 2019 Mar 21;19(6):1387. doi: 10.3390/s19061387 (PMC6470879; doi:10.3390/s19061387)
Supplement: Supplementary file 1 [file sensors-19-01387-s001.zip › Supplementary_Material/SI-3_q_Values/SM3_Notes.rtf]

Supplementary_Material_3 - SM3q_values is a pdf file representing the q-values used to correct the p-values after performing False Discovery Rate.
