# Supplementary material for: IoT and Engagement in the Ubiquitous Museum
Source: Sensors (Basel). 2019 Mar 21;19(6):1387. doi: 10.3390/s19061387 (PMC6470879; doi:10.3390/s19061387)
Supplement: Supplementary file 1 [file sensors-19-01387-s001.zip › Supplementary_Material/SI-4.pdf]

Supplementary Information 4 (SI-4)

We tested for differences between all 91 pairs distribution of length-of-visit. The Table below shows the p-values and q-values for significant differences after using Mann-Whitney-Wilcoxon Statistics corrected with False Discovery Rate for multiple tests.

Most of the significant differences describe that the two rooms with lowest (gray) and highest (red) activity are significantly different from most other rooms in blue.

Red links indicate pairs of rooms that are significantly different involving one of the most active rooms.

Green links indicate pairs of rooms that are significantly different involving one of the least active rooms.

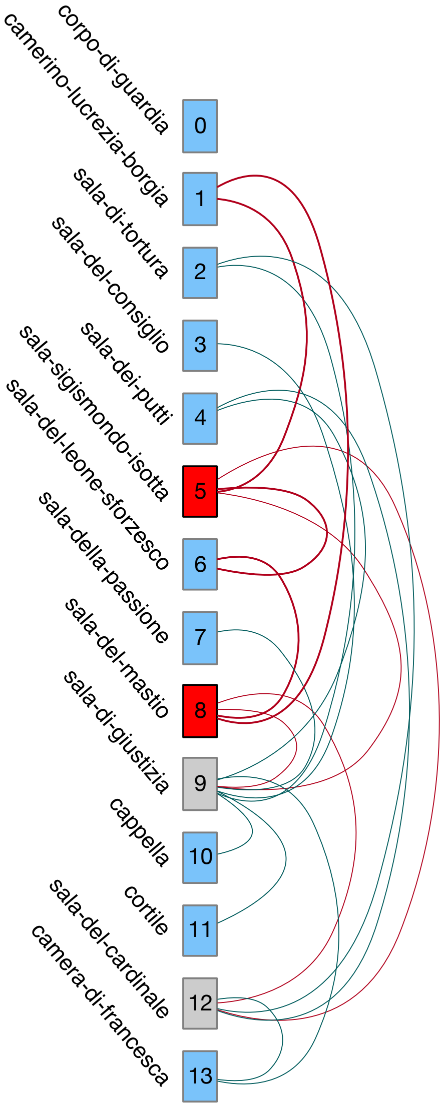

Test	Ri	Rj	P-value	Rank	Q-value
**0**	5	9	6.010146e-07	1	0.002747
**1**	8	9	1.531985e-06	2	0.005495
**2**	5	12	3.554627e-06	3	0.008242
**3**	8	12	9.576308e-06	4	0.010989
**4**	9	13	2.361519e-05	5	0.013736
**5**	4	9	3.973045e-05	6	0.016484
**6**	5	6	1.288692e-04	7	0.019231
**7**	12	13	1.296792e-04	8	0.021978
**8**	2	9	1.556860e-04	9	0.024725
**9**	4	12	1.998824e-04	10	0.027473
**10**	9	11	2.953695e-04	11	0.030220
**11**	6	8	3.003496e-04	12	0.032967
**12**	9	10	5.858513e-04	13	0.035714
**13**	1	5	6.898575e-04	14	0.038462
**14**	3	9	7.724352e-04	15	0.041209
**15**	2	12	8.024892e-04	16	0.043956
**16**	7	9	8.808666e-04	17	0.046703
**17**	1	8	1.177255e-03	18	0.049451
