# Supplementary material for: IoT and Engagement in the Ubiquitous Museum
Source: Sensors (Basel). 2019 Mar 21;19(6):1387. doi: 10.3390/s19061387 (PMC6470879; doi:10.3390/s19061387)

Supplementary Information 5 (SI-5). PCA scores for the individuals.

Visitor/ PC	PC.1	PC.2	PC.3	PC.4
1	1.32	0.03	-0.87	-0.05
2	-1.29	-0.48	0.27	-0.39
3	0.82	0.19	-0.87	0.13
4	-1.24	-0.74	0.16	-0.42
5	1.70	-0.16	-0.74	0.20
6	-1.12	-0.87	0.25	-0.47
7	-1.22	-0.84	0.14	-0.42
8	-0.78	-0.40	0.47	-0.15
9	-1.27	-0.58	0.22	-0.40
10	-1.17	-0.71	0.04	-0.36
11	-1.23	-0.79	0.15	-0.42
12	-1.23	-0.81	0.16	-0.42
13	-0.74	-0.77	-0.06	-0.56
14	6.84	1.41	-3.70	1.10
15	1.14	0.38	-0.13	-0.08
16	3.10	0.76	0.01	0.94
17	-1.12	-0.87	0.25	-0.47
18	-0.56	-0.58	0.32	-0.01
19	0.35	0.37	-0.36	0.26
20	0.51	-0.66	-0.12	1.29
21	0.26	-0.13	-0.21	-0.46
22	-1.78	7.64	-3.86	-0.98
23	-1.16	-1.20	2.05	1.79
24	-1.05	-0.83	0.10	-0.43
25	-0.83	-0.67	0.31	-0.13
26	-1.12	-0.41	0.28	-0.45
27	-1.18	-0.67	-0.02	-0.38
28	-1.11	-0.53	-0.01	0.37
29	-1.07	-0.78	0.20	-0.13
30	-0.80	-0.85	0.25	-0.14
31	-0.93	-0.66	-0.13	0.29
32	-3.19	10.69	3.27	0.98
33	0.74	-0.81	0.38	6.18
34	10.54	1.41	3.90	-1.88
35	0.48	-0.40	-0.45	0.47
36	-1.15	-0.88	0.19	-0.28
37	-1.24	-0.74	0.15	-0.42
38	-1.23	-0.79	0.18	-0.41
39	1.06	-0.09	-0.26	0.23
40	-0.08	-0.70	0.61	0.44
41	0.91	0.08	-1.00	0.36
42	-0.91	-0.63	-0.40	0.06
43	-1.25	-0.70	0.21	-0.40
44	-1.24	-0.74	0.19	-0.41
45	0.39	-0.29	-0.93	-0.20
46	2.69	-0.02	-0.08	-0.24
47	0.57	-0.51	-0.17	-0.89
48	-0.38	-0.25	0.19	-0.72
49	1.32	0.20	0.00	-0.59
50	-0.78	0.00	-0.04	-0.33
51	0.69	0.39	-0.47	-0.57

The table on the left contains the individual scores for the 51 individuals obtained from the PCA performed on the original dataset. Notice that the visit pattern of a given individual is approximated by the linear combination of orthogonal principal components (PCs).

The stacked bar chart below allows for easy inspection of how individual scores are generally not completely skewed to a single PC but instead are generally a combination of different PCs. The most skewed individual is ind=33 described in the main text.

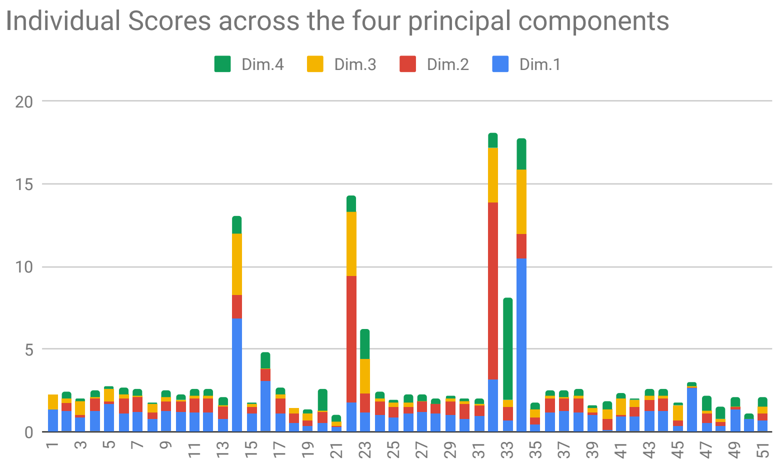

Supplement: Supplementary file 1 [file sensors-19-01387-s001.zip › Supplementary_Material/SI-5.pdf]
