# Supplementary material for: IoT and Engagement in the Ubiquitous Museum
Source: Sensors (Basel). 2019 Mar 21;19(6):1387. doi: 10.3390/s19061387 (PMC6470879; doi:10.3390/s19061387)
Supplement: Supplementary file 1 [file sensors-19-01387-s001.zip › Supplementary_Material/SI-6.pdf]

Supplementary Information 6 (SI-6)
Statistical significance of PCA factor (PC) loadings.

This table contains the loadings p>0.3 in the original PCA, the p-value from the Shapiro_Wilks test for strength of correlation between loadings and corresponding PC; the confidence internal 95% for the bootstrap sample mean with R=1000 and finally the result of a loading stability criterion defined as positive by either having the original loading in the obtained CI or by the minimum value of the CI being greater than the threshold p=0.3. All positive cases fall into the first criterion, except the only high loading for PC4 which in the bootstrap distribution has a lower median which is still above 0.3.

Variable	Loading	p-Value	Median CI 95%	Stable?
**PC-1**				
corpo-di-guardia	0.86	1.100000e-14	(0.86, 0.87)	yes
camerino-lucrezia-borgia	0.79	8.390000e-12	(0.75, 0.79)	yes
sala-del-leone-sforzesco	0.88	7.280000e-17	(0.88, 0.89)	yes
sala-della-passione	0.88	1.670000e-16	(0.84, 0.88)	yes
cappella	0.83	8.700000e-14	(0.79, 0.83)	yes
cortile	0.79	1.830000e-11	(0.77, 0.79)	yes
camera-di-francesca	0.54	5.740000e-05	(0.51, 0.54)	yes
**PC-2**				
sala-di-tortura	0.94	9.610000e-24	(0.91, 0.94)	yes
sala-del-consiglio	0.80	5.950000e-12	(0.67, 0.81)	yes
sala-sigismondo-isotta	0.96	2.790000e-27	(0.94, 0.97)	yes
sala-del-mastio	0.97	2.530000e-29	(0.95, 0.97)	yes
sala-di-giustizia	0.57	1.560000e-05	(0.52, 0.58)	yes
**PC-3**				
corpo-di-guardia	0.35	1.330000e-02	(-0.13, -0.11)	no
sala-del-consiglio	0.39	5.030000e-03	(0.03, 0.04)	no
sala-di-giustizia	-0.44	1.210000e-03	(-0.03, -0.01)	no
cortile	0.44	1.530000e-03	(-0.15, -0.12)	no
sala-del-cardinale	0.30	3.520000e-02	(-0.005, -0.01)	no
camera-di-francesca	-0.67	1.490000e-07	(0.08, 0.13)	no
**PC-4**				
sala-dei-putti	0.91	1.780000e-02	(0.46, 0.52)	yes
