# Supplementary figures and images for: IoT and Engagement in the Ubiquitous Museum
Source: Sensors (Basel). 2019 Mar 21;19(6):1387. doi: 10.3390/s19061387 (PMC6470879; doi:10.3390/s19061387)

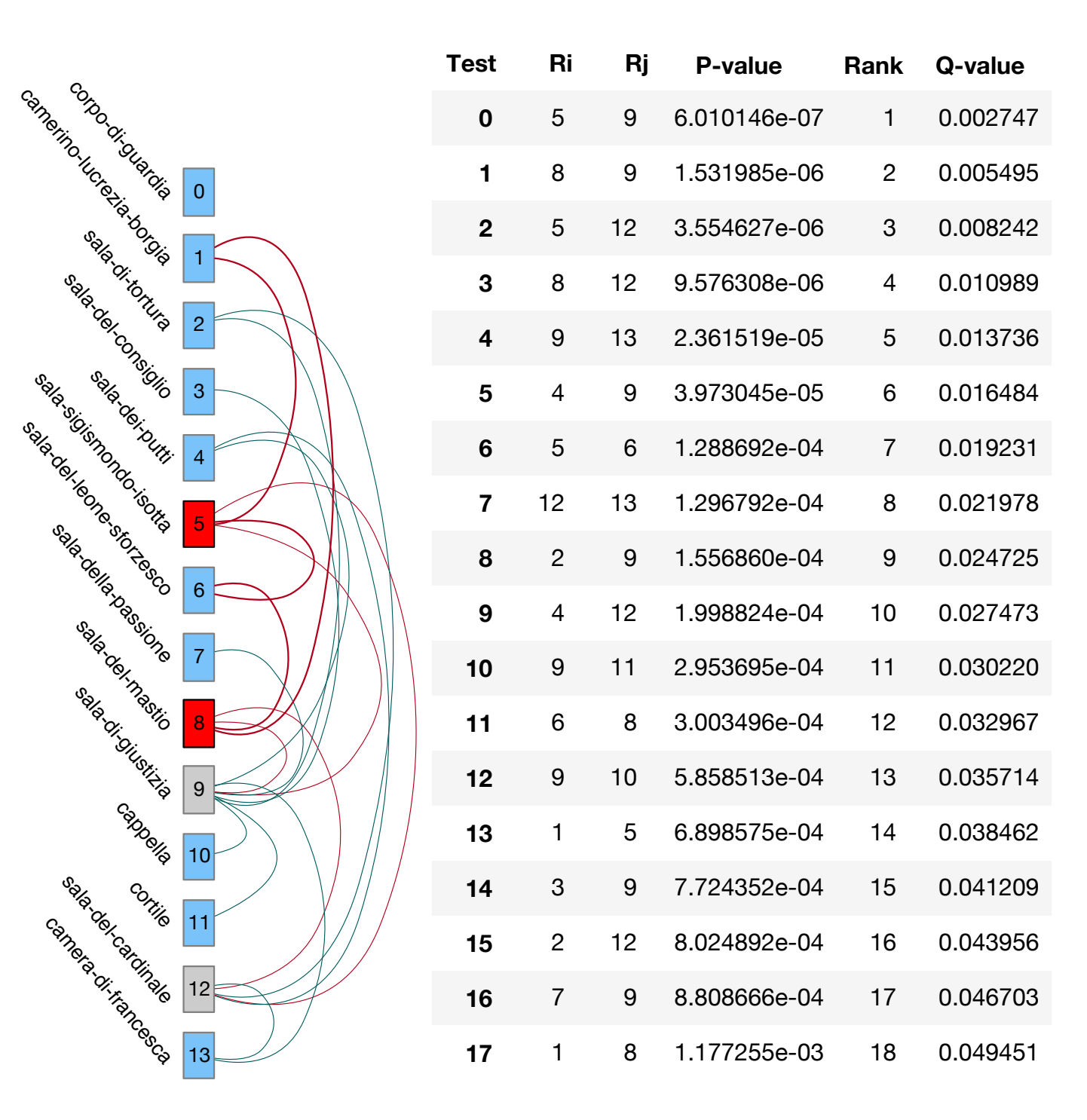

Supplement: Supplementary file 1 [file sensors-19-01387-s001.zip › Supplementary_Material/SI-3_q_Values/q_values.pdf]
